# HAL: a hierarchical format for storing and analyzing multiple genome alignments

**DOI:** 10.1093/bioinformatics/btt128

**Published:** 2013-03-16

**Authors:** Glenn Hickey, Benedict Paten, Dent Earl, Daniel Zerbino, David Haussler

**Affiliations:** ^1^Center for Biomolecular Science and Engineering, University of California Santa Cruz, Santa Cruz CA 95064, USA and ^2^Howard Hughes Medical Institute, University of California, Santa Cruz, CA 95064, USA

## Abstract

**Motivation:** Large multiple genome alignments and inferred ancestral genomes are ideal resources for comparative studies of molecular evolution, and advances in sequencing and computing technology are making them increasingly obtainable. These structures can provide a rich understanding of the genetic relationships between all subsets of species they contain. Current formats for storing genomic alignments, such as XMFA and MAF, are all indexed or ordered using a single reference genome, however, which limits the information that can be queried with respect to other species and clades. This loss of information grows with the number of species under comparison, as well as their phylogenetic distance.

**Results:** We present HAL, a compressed, graph-based hierarchical alignment format for storing multiple genome alignments and ancestral reconstructions. HAL graphs are indexed on all genomes they contain. Furthermore, they are organized phylogenetically, which allows for modular and parallel access to arbitrary subclades without fragmentation because of rearrangements that have occurred in other lineages. HAL graphs can be created or read with a comprehensive C++ API. A set of tools is also provided to perform basic operations, such as importing and exporting data, identifying mutations and coordinate mapping (liftover).

**Availability:** All documentation and source code for the HAL API and tools are freely available at http://github.com/glennhickey/hal.

**Contact:** hickey@soe.ucsc.edu or haussler@soe.ucsc.edu

**Supplementary information:** Supplementary data are available at *Bioinformatics* online.

## 1 INTRODUCTION

A DNA (or protein) sequence alignment groups together positions in the sequences they contain that are homologous (related by descent). Positions within the same sequence can be homologous via duplication events. The multiple alignment problem is NP-hard, but tools have been developed to produce large (tens of thousands of sequences) (Notredame, 2007) accurate alignments, provided the input sequences are relatively short and conserved, such as gene exons. Whole-genome alignment is much more difficult not only because of the increased length of the input but also because of the presence of large spans of non-conserved sequence (Blanchette *et al.*, 2004; Paten *et al.*, 2011). Changes because of large-scale rearrangement events, such as inversions, segmental duplications and transpositions, must also be taken into account in addition to point mutations and small insertions and deletions (indels).

These challenges of creating whole-genome alignments carry over to their representation and analysis. Gapped matrices that are traditionally used for gene alignments become fragmented into blocks by rearrangements or excessive divergence. These blocks are stored by current formats as ASCII lines containing coordinates and DNA strings for each sequence in the alignment. As blocks can only be ordered with respect to a single reference row, performing disk-based queries using non-reference coordinates is extremely inefficient because of fragmentation, even if external indexes were to be constructed for them. The hierarchical alignment (HAL) graph structure and tool set described later in the text were designed to address this issue, while adding support for file compression.

## 2 METHODS

### 2.1 HAL format

HAL’s design was guided by two observations: (i) breakpoint graphs are the most natural way of representing genome rearrangements (Pevzner and Tesler, 2003) and (ii) progressive alignment (based on a phylogenetic decomposition) has been the most successful heuristic for multiple sequence alignment (Notredame, 2007) and is likely to remain so for whole-genome alignment. A HAL graph, therefore, decomposes a multiple alignment into a set of pairwise alignments, which are represented as breakpoint graphs. Each pairwise alignment corresponds to a branch of a rooted phylogenetic tree. In the absence of a tree, a reference genome can be used as a root with all other genomes as leaves.

**Fig. 1.**
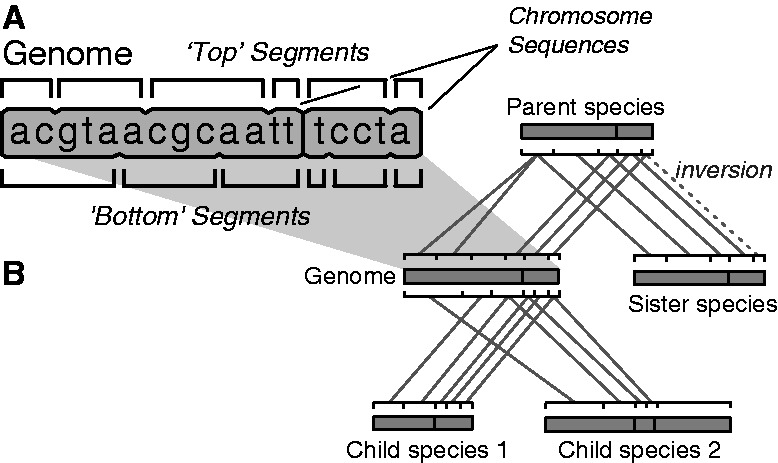
(**A**) A single genome as represented in HAL. Two sequences are stored in an array of DNA characters and are segmented with respect to its parent (top segments) and children (bottom segments). (**B**) The same genome in the context of HAL graph of five genomes. The dashed edge corresponds to an inversion event

A genome in HAL is represented by up to three arrays (Fig. 1A): a *sequence array*, a *top segment array* if the genome has an ancestor in the tree and a *bottom segment array* if the genome has one or more descendants in the tree. For each branch in the phylogenetic tree, edges in the HAL graph connect bottom segments from the ancestral genome to top segments in the descendant genome (Fig. 1B). These edges define the pairwise alignments between the ancestral genome and each of its descendants. The amount of segmentation along a branch is, therefore, determined by the number of unique breakpoints in these pairwise alignments, including those induced by indels. Each top (resp. bottom) segment *S* is assigned a *parse edge* connecting it to the bottom (resp. top) segment of the same genome that overlaps the first base of *S* on the DNA sequence. Paralogs (duplicated) regions of the genome are represented by sets of top segments that share an ancestor. Inversions are represented as flags on edges between segments. Chromosomes or scaffolds are represented as contiguous subranges of the genome.

Provided the graph is in memory, all segments and DNA bases can be looked up by their array index, and all edges can be traversed, in *O*(1) time. Locating the segment that contains a particular DNA position within a given chromosome requires 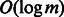 time, where *m* is the number of segments. Heuristics are used to further speed this operation up in practice. An arbitrary amount of metadata can be added to each genome. Missing data are stored as segments of ‘N’ characters with no edges. Supplementary Sections S1 and S3 describe how HAL offers similar compression to gzip and vastly reduced query times when compared with Multiple Alignment Format (MAF).

### 2.2 HAL API

A comprehensive C++ API is provided to create and query HAL files, which are presently stored in Hierarchical Data Format (HDF5). HDF5 is a longtime standard, supported on all major platforms, for storing large matrix data and is optimized for efficient indexing, caching and compression (The HDF5 Group, 2000–2010). Data within HDF5 can be quickly and randomly indexed, and decompression and caching is abstracted from the user. This allows for efficient external memory algorithm design, a requirement for multi-genome analysis. We note that the different back ends could be added in the future.

HAL graphs are accessed by *segment iterators*, which traverse the graph through its native structure of segments and edges, and *column iterators*, which dynamically transpose the graph (or desired subgraph) to a traditional matrix block/format to iterate across alignment columns.

### 2.3 HAL tools

A set of command line utilities, summarized in Table 1, are provided to create and analyze multiple genome alignments in HAL. Importers are provided for UCSC’s MAF, which is a standard with its own rich set of filters and converters (ex. to FASTA) (Blanchette *et al.*, 2004) and Cactus (Paten *et al.*, 2011), which has been designed specifically to output HAL. MAF files can be quickly produced from HAL graphs for given subgraphs with respect to arbitrary references to be compatible with existing browsers and tools. The memory usage of each tool is configurable via its command line options.

**Table 1.** HAL tools summary

Tool	Description
halStats	Print summary statistics of HAL file
halSummarizeMutations	Print mutation summary for given subgraph
halBranchMutations	Generate BED file(s) of mutations for a branch
halLiftover	Map BED coordinates between genomes
hal2maf/maf2hal	Convert to and from MAF
cactus2hal	Convert from Cactus

Mutations can be identified along branches and output to tab delimited annotation files using the *halBranchMutations* tool. A cycle decomposition of the breakpoint graph structure allows rearrangements, such as duplications, inversions and transpositions to be reported in addition to substitutions, insertions and deletions. Small indels (determined by a provided threshold) can be nested within larger rearrangements to avoid overcounting in these cases. Patterns of conservation within a target sequence can be aggregated using the *halLiftover* tool, which maps coordinates in a BED file to an arbitrary target in the alignment. This utility provides a general strategy to efficiently liftover and project any comparative genomics information into the coordinate system of any reference genome. Excellent software packages are available for sorting, combining and querying BED files (Quinlan and Hall, 2010; Neph *et al.*, 2012) and can be combined with the aforementioned tools to create powerful analysis pipelines for multiple genome alignments.

## 3 CONCLUSION

We have presented HAL, a data format, API and set of tools for storing and analyzing genome alignments and ancestral reconstructions. The key features of HAL are its indexing, which allows fast coordinate mapping between arbitrary subsets of genomes, and its graph structure, which facilitates analysis of genome rearrangements, as well as modular decomposition into clades. The compression and chunking capabilities of HDF5 are leveraged to keep I/O and memory usage to a minimum. All of these properties, in particular the ability to parallelize by clade, will be necessary for alignments that arise from current large-scale sequencing projects, such as Genome 10K (Haussler *et al.*, 2009) (more details in Supplementary Section S2).

## ACKNOWLEDGEMENT

We thank the reviewers for their valuable suggestions, as well as our sources of funding.

*Funding*: California Institute for Quantitative Biosciences (to G.H.). A Data Analysis Center for the Encyclopedia of DNA Elements 5U01HG004695 (NHGRI/NIH), and gift funds from Dr and Mrs Gordon Ringold (to B.P.). Howard Hughes Medical Institute (to D.H.).

*Conflict of Interest*: none declared.

## Supplementary Material

Supplementary Data

## References

[btt128-B1] Blanchette M (2004). Aligning multiple genomic sequences with the threaded blocks*et al.*gner. Genome Res..

[btt128-B2] Haussler D (2009). Genome 10k: a proposal to obtain whole-genome sequence for 10 000 vertebrate species. J. Hered..

[btt128-B3] Neph S (2012). Bedops: high-performance genomic feature operations. Bioinformatics.

[btt128-B4] Notredame C (2007). Recent evolutions of multiple sequence alignment algorithms. PLoS Comput. Biol..

[btt128-B5] Paten B (2011). Cactus: algorithms for genome multiple sequence alignment. Genome Res..

[btt128-B6] Pevzner P, Tesler G (2003). Genome rearrangements in mammalian evolution: lessons from human and mouse genomes. Genome Res..

[btt128-B7] Quinlan AR, Hall IM (2010). Bedtools: a flexible suite of utilities for comparing genomic features. Bioinformatics.

[btt128-B8] The HDF5 Group (2000–2010). Hierarchical data format version 5.

