# Supplementary material for: HAL: a hierarchical format for storing and analyzing multiple genome alignments
Source: Bioinformatics. 2013 Mar 16;29(10):1341–2. doi: 10.1093/bioinformatics/btt128 (PMC3654707; doi:10.1093/bioinformatics/btt128)
Supplement: Supplementary Data [file supp_29_10_1341__index.html]

HAL: a hierarchical format for storing and analyzing multiple genome alignments — HAL: a hierarchical format for storing and analyzing multiple genome alignments — Supplementary Data 

# HAL: a hierarchical format for storing and analyzing multiple genome alignments

## Supplementary Data

files

**Files in this Data Supplement:**

- Supplementary Data - pdf file
