# Supplementary material for: HAL: a hierarchical format for storing and analyzing multiple genome alignments
Source: Bioinformatics. 2013 Mar 16;29(10):1341–2. doi: 10.1093/bioinformatics/btt128 (PMC3654707; doi:10.1093/bioinformatics/btt128)
Supplement: Supplementary Data [file supp_btt128_hickey_hal_supplement3.pdf]

Supplemental Information for

HAL: A Hierarchical Format for Storing and Analyzing Multiple Genome Alignments

by Glenn Hickey, Benedict Paten, Dent Earl, Daniel Zerbino, and David Haussler

S1. Cactus 20 Drosophila Progressive Genome Alignment

Twenty drosophila genome assemblies of sizes ranging between 100 and 200 megabases were obtained from the Alignathon website (credits, details, and downloads at http://compbio.soe.ucsc.edu/alignathon/flies.html). A multiple genome alignment and ancestral reconstruction were generated using Progressive Cactus (manuscript in preparation, beta version available at https://github.com/glennhickey/progressiveCactus). The alignment of the 20 genomes as well as the 19 inferred ancestral genomes were stored in MAF (http://genome.ucsc.edu/FAQ/FAQformat.html - format5), gzipped MAF, and HAL format. The sizes of the resulting files are listed in Figure S1, which shows that the HAL graph is of comparable size to the gzipped MAF file, nearly ten times smaller than the uncompressed MAF.

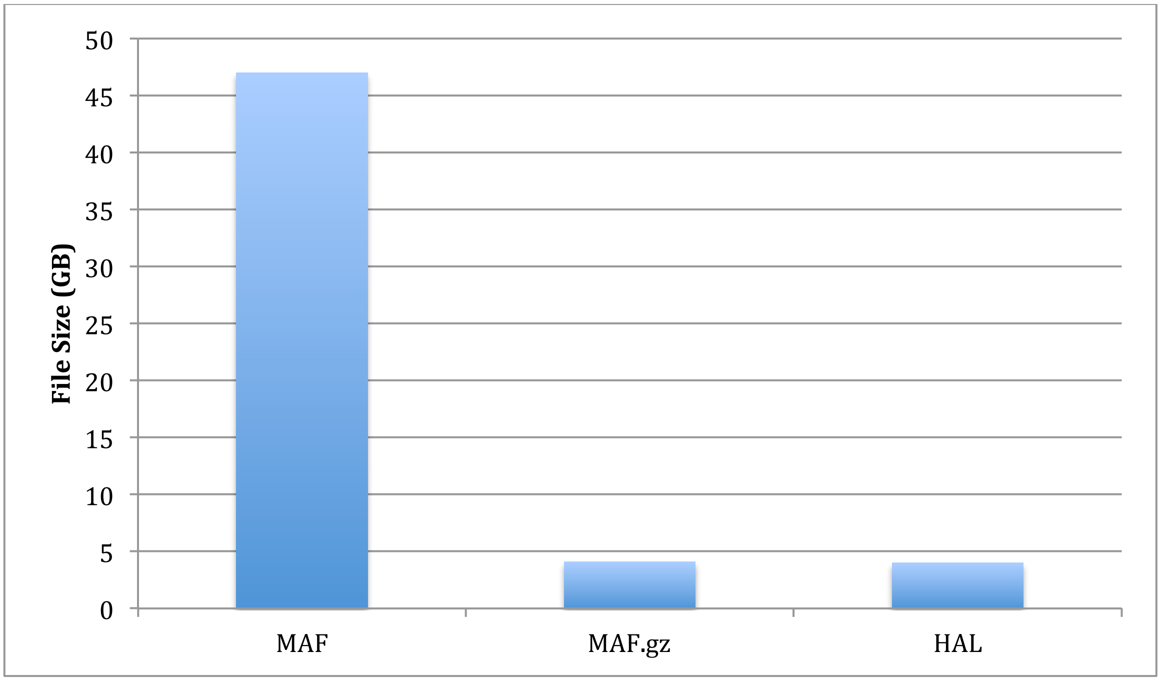

Figure S1: Drosophila Progressive Alignment Size

Scanning through the entire alignment and counting all substitutions is a simple way to benchmark file access without any regards to indexing. The HAL API contains tools to do perform this scan on both MAF (`mafMutations`) and HAL (`halSummarizeMutations`). The time required to perform this scan on the MAF and HAL versions of the drosophila alignment on a single Intel Xeon x7560 2.27Ghz core using under 4G RAM is reported in Figure S2. Despite being compressed, the HAL alignment can still be scanned more quickly than the text-based MAF. The advantage becomes more noticeable when performing a partial query, as shown in the HAL (sim vs. sec, d=2) column of Figure S2. This is the time taken to count all substitutions between the first million bases on chromosome 2L of *drosophila simulans* (droSim1) and aligned sites within *drosophila sechellia* (droSec1), which are separated by two branches. In the MAF file, which is indexed on *melanogaster*, this query can only be achieved by scanning the entire file. In contrast, HAL tools can be used to perform this query much more efficiently.

```
echo droSim1.chr2L 0 1000000 > droSim1_query.bed
prevGenome = droSim1
```

```
prevBed = droSim1_query.bed
for x in `halStats 20flys1000.hal --path droSim1,droSec1`; do\
      halLiftover 20flys.hal $prevGenome $prevBed $x ${x}.bed\
      halBranchMutations 20flys.hal $x --snpFile\
            ${x}_snp.bed;
      prevGenome = $x
      prevBed = ${x}.bed
      done
wc –l *_snp.bed
```

The above query was repeated using *drosophila yakuba*, *drosophila pseudoobscura*, and *drosophila virilis* as targets, with the respective results displayed in the last three columns of Figure S2. These genomes cover a range of distances on the tree, from 5 branches for *yakuba* to 14 (the diameter of the tree) for *virilis*. The running time of these queries is, as expected, proportional to the number of branches that must be analyzed.

We note that the above code can be trivially parallelized. Queries on subregions can also be performed by generating MAF files, if desired:

```
hal2maf 20flys.hal out.maf --refGenome droSim1 –refSequence\
      droSim1_query.bed --start 0 --length 1000000 –targetGenomes\
      droSec1
mafMutations out.maf
```

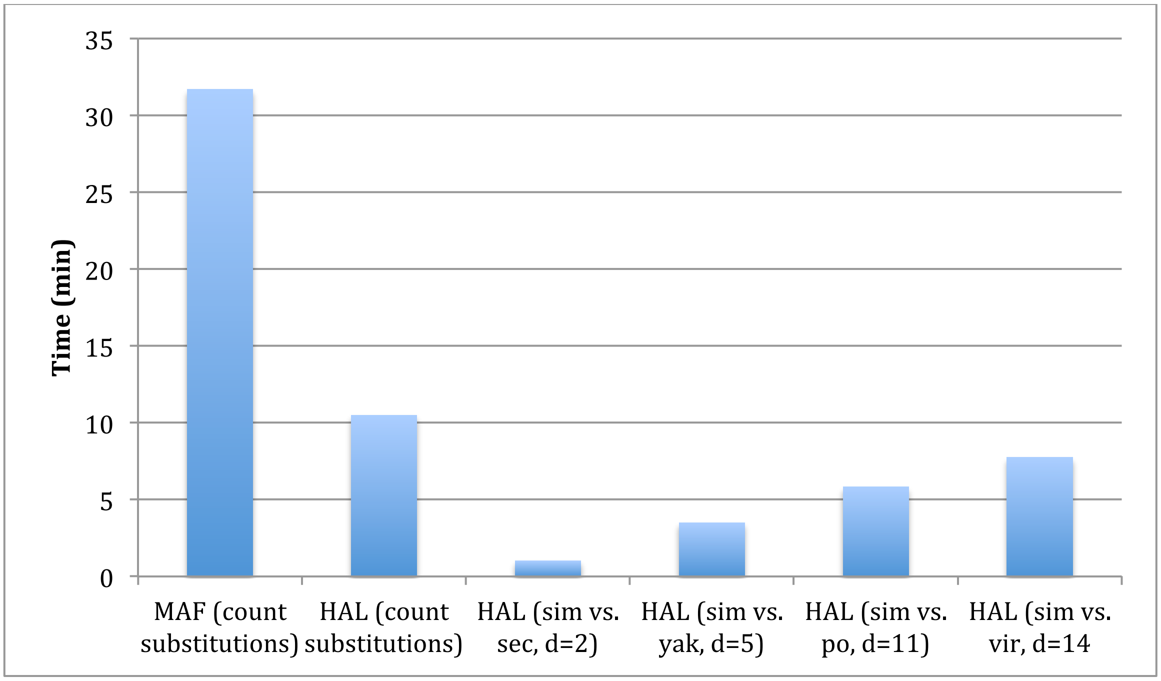

Figure S2: Drosophila Progressive Alignment Query Time

S2. Projection to 10000 Vertebrate Genome Alignment

The range of branch lengths on the drosophila tree (from 0.006 – 0.26 substitutions per site), as well as the diversity of assembly qualities, makes it a good candidate to extrapolate, albeit crudely, trends for the eventual progressive alignment and reconstruction of the vertebrate phylogeny targeted by the Genome 10k project (www.genome10k.org). Assuming the average vertebrate genome is 20x larger than a fly genome (in the following section, we verify that HAL scales in this dimension using Multiz alignments), we use a simple linear scale to estimate alignment the Genome 10K HAL alignment (10000 genomes and 9999 ancestors) size to be 40TB, over 400TB smaller than MAF. Further gains in storage footprint could theoretically be obtained by using a wider (degree > 2) tree topology, requiring fewer ancestral genomes.

We use a similar strategy to infer the running times associated with counting substitutions in the alignment as shown in Figure S3. The time to analyze an

arbitrary clade of twenty species (rooted subtree with 39 nodes) in HAL is estimated as 20x the time to scan all of the flies. We can make this assumption because of the modular structure of the HAL graph. The same analysis on a non-modular format such as MAF would be proportional to the total scan time, thousands of hours longer. We claim that the decomposition that HAL provides is therefore necessary for the analysis of large genome alignments in reasonable timeframes, as it provides a principled framework for parallelization.

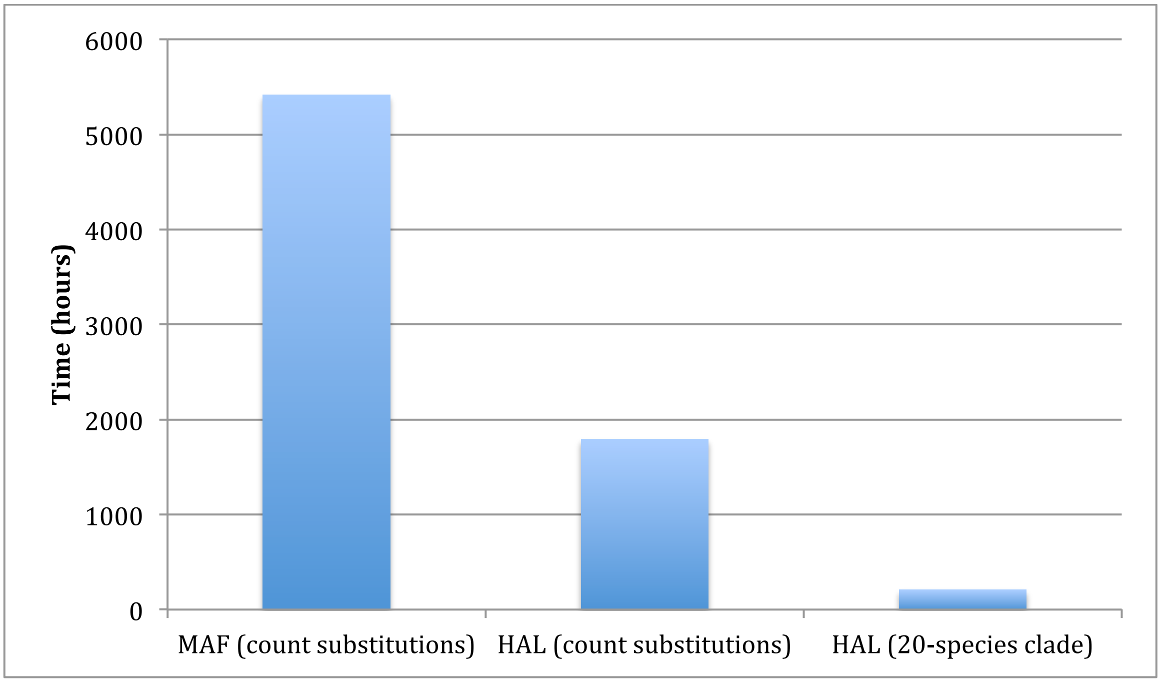

Figure S3: Genome10k Progressive Alignment Query Time Estimates

In general, the analysis of a set of species in a HAL format will be dependent on the size of their spanning tree. In this sense, a clade is a best-case scenario since the number of internal branches is minimal. Still as shown in Figure S2, we can expect gains when analyzing distant sets of species when compared to the entire tree.

S3. Multiz Three Vertebrate Alignment

To benchmark how HAL scales to full vertebrate genomes, we began by extracting a three-way alignment between mouse, rat, and kangaroo rat from the 60-way MultiZ alignment to mouse available on the UCSC Genome Browser:

```
rsync -avz --progress \
rsync://hgdownload.cse.ucsc.edu/goldenPath/mm10/multiz60way/ ./
for i in *.maf.gz; do gzip –c –d $i >> mouse60.maf; done
mafFilter[1] mouse60.maf --includeSeq mm10,dipOrd1,rn5 > mouse3.maf
maf2hal mouse3.maf mouse3.hal
```

Since the MultiZ alignment is reference-based, we use a HAL star tree with the reference (mouse) as the ancestral node to represent it. While the semantics between a reference species and ancestor a very different, in terms of the HAL format they can be represented equivalently. The alignment sizes and scan times are shown in Figure S4 and Figure S5, respectively. The relative performance of the HAL alignments in this case is lower than the drosophila alignments, with the HAL file being significantly larger than the gzipped MAF and taking longer to scan. This is due to low coverage of the Multiz reference alignments: only 67% and 19% of the rat and kangaroo rat genomes are aligned to mouse, respectively, and stored in the MAF file. In contrast, the HAL alignment stores a position for every base in each genome. Finally, even in this case where HAL is storing more information, its indexing allows partial queries to be performed much more efficiently. For example, printing all (~11M) substitutions between rat chromosome 20 and mouse (last column of Figure S5) is still over an order of magnitude faster in HAL:

```
echo chr20 0 57791882 > rn5.chr20.bed
halBranchMutations mouse3.hal --refTargets rn5.chr20.bed \
--snpFile rn5.chr20_snp.bed
```

[1] Available at github.com/dentearl/mafTools/

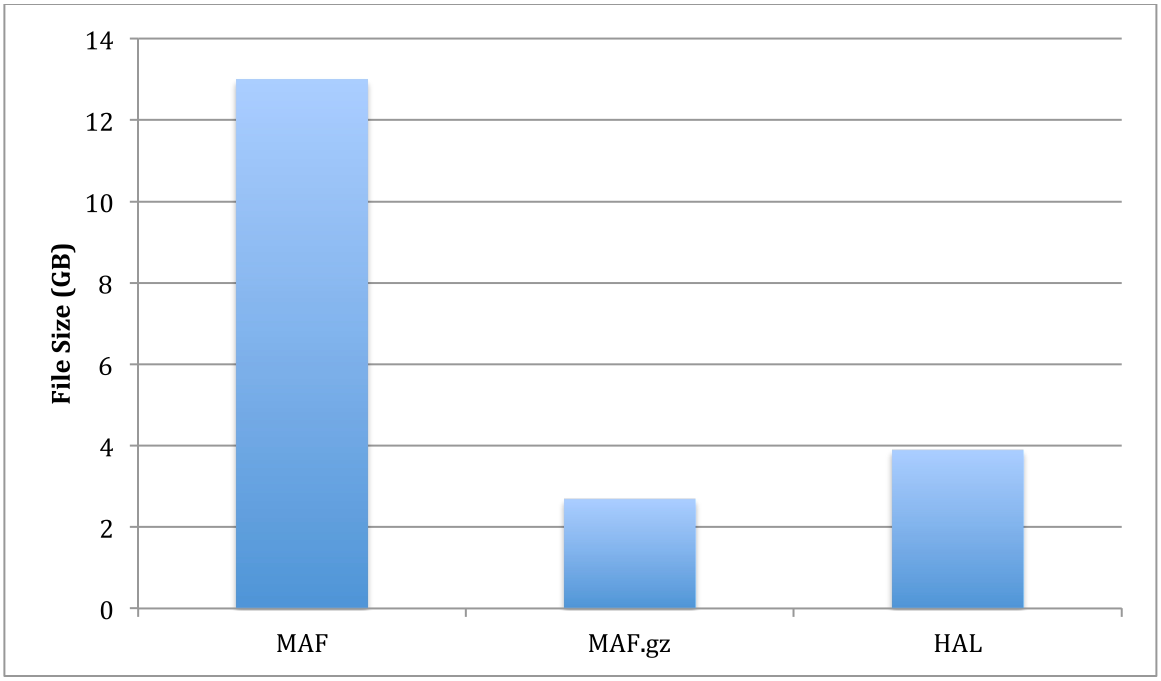

Figure S4: Multiz 3-Vertebrate Alignment Size

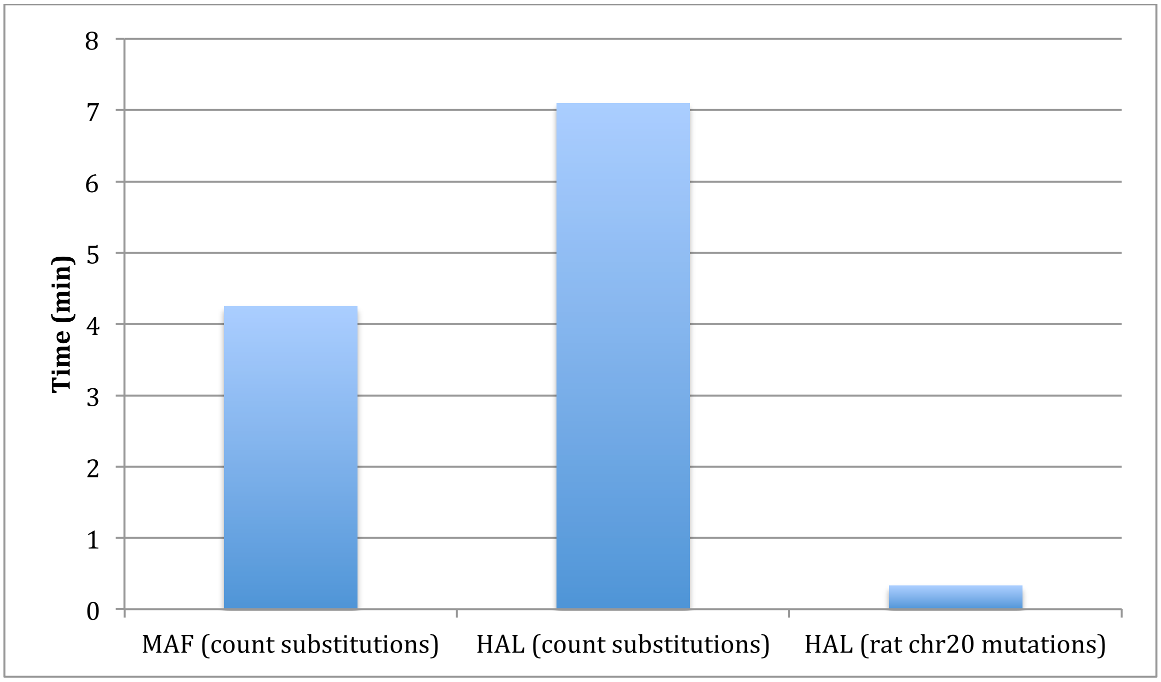

Figure S5: Multiz 3-Vertebrate Alignment Query Time

S4. Acknowledgements

We thank the reviewers for their valuable suggestions, as well as our sources of funding: Glenn Hickey was funded by the California Institute for Quantitative Biosciences. Benedict Paten was funded by A Data Analysis Center for the Encyclopedia of DNA Elements 5U01HG004695 (NHGRI/NIH), and gift funds from Dr. and Mrs. Gordon Ringold. David Haussler was funded by Howard Hughes Medical Institute.
